# The Spread of NDM-1 and NDM-7-Producing *Klebsiella pneumoniae* Is Driven by Multiclonal Expansion of High-Risk Clones in Healthcare Institutions in the State of Pará, Brazilian Amazon Region

**DOI:** 10.3390/antibiotics10121527

**Published:** 2021-12-14

**Authors:** Yan Corrêa Rodrigues, Amália Raiana Fonseca Lobato, Ana Judith Pires Garcia Quaresma, Lívia Maria Guimarães Dutra Guerra, Danielle Murici Brasiliense

**Affiliations:** Bacteriology and Mycology Section, Evandro Chagas Institute (SABMI/IEC), Ananindeua 67030-000, PA, Brazil; yan.13@hotmail.com (Y.C.R.); amalialobato@iec.gov.br (A.R.F.L.); anaquaresma@iec.gov.br (A.J.P.G.Q.); liviadutra@iec.gov.br (L.M.G.D.G.)

**Keywords:** *Klebsiella pneumoniae*, Gram-negative bacteria, antimicrobial resistance, multi-drug resistance, carbapenemase, molecular epidemiology, CC258, CC15, ST392

## Abstract

Carbapenem resistance among *Klebsiella pneumoniae* isolates is often related to carbapenemase genes, located in genetic transmissible elements, particularly the *bla*_KPC_ gene, which variants are spread in several countries. Recently, reports of *K. pneumoniae* isolates harboring the *bla*_NDM_ gene have increased dramatically along with the dissemination of epidemic high-risk clones (HRCs). In the present study, we report the multiclonal spread of New Delhi metallo-beta-lactamase (NDM)-producing *K. pneumoniae* in different healthcare institutions in the state of Pará, Northern Brazil. A total of 23 NDM-producing isolates were tested regarding antimicrobial susceptibility testing features, screening of carbapenemase genes, and genotyping by multilocus sequencing typing (MLST). All *K. pneumoniae* isolates were determined as multidrug-resistant (MDR), being mainly resistant to carbapenems, cephalosporins, and fluoroquinolones. The *bla*_NDM-7_ (60.9%—14/23) and *bla*_NDM-1_ (34.8%—8/23) variants were detected. MLST genotyping revealed the predomination of HRCs, including ST11/CC258, ST340/CC258, ST15/CC15, ST392/CC147, among others. To conclude, the present study reveals the contribution of HRCs and non-HRCs in the spread of NDM-1 and NDM-7-producing *K. pneumoniae* isolates in Northern (Amazon region) Brazil, along with the first detection of NDM-7 variant in Latin America and Brazil, highlighting the need for surveillance and control of strains that may negatively impact healthcare and antimicrobial resistance.

## 1. Introduction

*Klebsiella pneumoniae* is among the major pathogens causing healthcare-related infections (HAIs) and outbreaks in several healthcare institutions. This is due to its antimicrobial resistance (AMR) and virulence traits, leading to the increasingly frequent reports of severe infections, poor prognosis outcomes, and limitation of antimicrobial therapy options [1,2].

The aggravating problem of AMR among *K. pneumoniae* isolates is commonly related to the spread of plasmid-borne resistance genes, including metallo-beta-lactamases (MβLs) and extended spectrum beta-lactamases (ESβLs). Carbapenem-resistant *K. pneumoniae* isolates (CR-Kp) are usually detected harboring the *bla*_KPC_ gene, with endemicity reported in Brazil and several other countries, while the dissemination of NDM-producing *K. pneumoniae* has increased in several regions, and mainly during the COVID-19 pandemic [3,4,5,6,7,8]. Such highly resistant-strains carrying resistance mechanisms are associated with so-called high-risk clones (HRCs), particularly to strains belonging to the clonal complex 258 (CC258) and CC15, which have been detected in multiclonal expansion at several Brazilian hospitals [9,10,11,12,13,14].

Even though other studies have demonstrated the spread of *bla*_KPC_ and *bla*_NDM_ by *K. pneumoniae* HRC strains across Brazil [12,15,16,17,18], critical knowledge gaps remain regarding this distribution and genetic background contributing to this dissemination in Northern Brazil. Herein, we report a genetic background mostly composed of HRCs, and the multiclonal spread of NDM-1 and NDM-7-producing *K. pneumoniae* in different healthcare institutions in the state of Pará, the Brazilian Amazon region.

## 2. Results

### 2.1. Bacterial Isolates and Susceptibility Characteristics

*K. pneumoniae* isolates were recovered from patients at nine different healthcare institutions in the region (H1-H9), of which 43.5% (*n* = 10) were hospitalized in intensive care unit (ICUs) and 56.5% (*n* = 13) in clinical wards (non-ICU settings). Clinical specimen of *K. pneumoniae* samples included: urine (*n* = 8), blood (*n* = 3), tracheal secretion (*n* = 3), rectal swab (*n* = 3), bronchoalveolar lavage (*n* = 1), abdominal abscess secretion (*n* = 1), wound secretion (*n* = 1), nasopharyngeal secretion (*n* = 1), soft tissue secretion (*n* = 1), and peritoneal fluid (*n* = 1) (Table 1).

Antimicrobial susceptibility testing (ATS) results demonstrated that the majority of *K. pneumoniae* isolates were non-susceptible to the tested antimicrobial classes and phenotypically classified as multi-drug resistant (MDR—possible XDR) (23/23—100.0%). Isolates exhibited a high frequency of resistance to carbapenems (ETP and MEM 23/23—100.0%; IMP 22/23 95.7%), 3rd/4th generation cephalosporins (CAZ, CRO and FEP 23/23—100.0%), and fluoroquinolones (CIP 21/23—91.3%). Differently, tested isolates were mainly susceptible to TGC (18/23—78.3%) and aminoglycosides (AMK 13/23—56.5%; GEN 12/23—52.2%). The mCIM/eCIM essays revealed that all of the isolates were carbapenemase-producing (mCIM-positive); however, three isolates were negative to EDTA inhibition (eCIM) (Table 2).

Molecular screening of carbapenemase genes showed that all *K. pneumoniae* isolates harbored the *bla*_NDM_ gene (23/23—100.0%), including 60.9% (14/23) defined as *bla*_NDM-7_ variant and 34.8% (8/23) as a *bla*_NDM-1_ variant. The definition of the bla_NDM_ subtype of one isolate (4.3%) could not be performed. Moreover, two isolates were detected (8.7%) co-harboring the *bla*_NDM-7_/*bla*_KPC-2_ genes, while the other two (8.7%) were the *bla*_NDM-1_/*bla*_KPC-2_ genes. The *bla*_IMP_, *bla*_VIM,_ and *bla*_OXA-48_ genes were not detected (Table 1 and Table 2).

### 2.2. Molecular Typing by Multilocus Sequencing Typing—MLST

**Figure 1:**
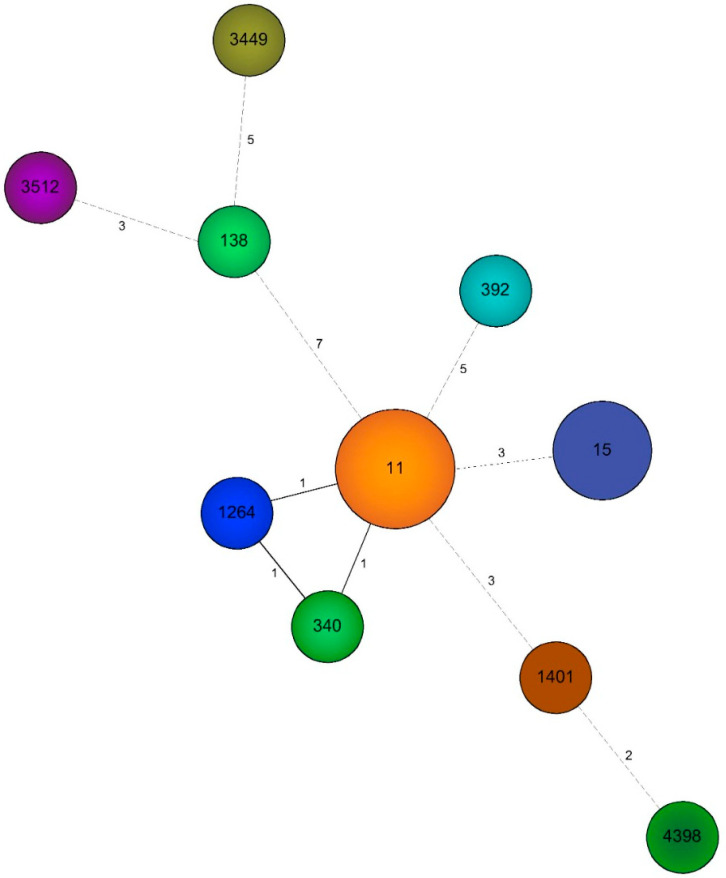
Minimum spanning tree (MST) of the 23 NDM-producing *K. pneumoniae* isolates genotyped by MLST. Each circle represents a different ST, and the size of the circle is proportional to the number of isolates related to the respective ST. The number next to the lines represents the number of loci variations between STs.

MLST genotyping and genetic relationship analysis revealed a genetic background mostly composed by HRCs, including nine sequence types (STs) associated with four clonal complexes (CCs), including: HRC ST11/CC258 (10/23—43.5%), HRC ST15/CC15 (5/23—21.7%), HRC ST340/CC258 (1/23—4.3%), HRC ST392/CC147 (1/23—4.3%), HRC ST1264/CC258 (1/23—4.3%), ST1401/CC1401(1/23—4.3%) and ST138/CC138 (1/23—4.3%); while ST3449 (1/23—4.3%) and ST3512 (1/23—4.3%) ST4398 (1/23—4.3%) were classified as singletons (Table 1 and Figure 1).

The bla_NDM-7_ variant was found to spread in seven healthcare institutions (H1, H2, H3, H5, H7, H8, and H9) mostly related to *K. pneumoniae* CC258 strains, including ST11 and ST1264, and non-HRCs. Oppositely, the dissemination of the bla_NDM-1_ variant was related to three STs (ST15, ST11, and ST340) at four hospitals (H2, H3, H4, and H6). Finally, the four isolates harboring both *bla*_NDM_ and *bla*_KPC_ genes were associated with the HRC ST11/CC258 circulating at H3 and H6 (Table 1).

## 3. Discussion

The emergence and spread of MDR CR-Kp strains have been reported worldwide and in Brazil, causing a critical impact on the increasing levels of antimicrobial resistance and leading to a public health crisis due to the limitation of antimicrobial therapy options, infection severity, and challenging spread control. Such strains are usually related to epidemic HRCs harboring plasmid-borne carbapenemases, particularly the *bla*_KPC_, and recently *bla*_NDM_, emphasizing the importance of epidemiological vigilance and recognizing medically relevant resistant features strains. The present study reports the spread of NDM-producing *K. pneumoniae* strains associated with HRCs in different healthcare institutions in the state of Pará, Brazilian Amazon region.

The resistance trend for 3rd/4th generation cephalosporins and carbapenems among *K. pneumoniae* isolates have been reported in several countries, and in Latin America, countries including Brazil, Argentina and Chile account for most of the cases [1,2,5]. Indeed, on recently reported data of national surveillance on antimicrobial resistance, *K. pneumoniae* isolated in adult and pediatric Brazilian ICUs presented resistance rates ranging 49.3% to 68.1% and 19.3% to 51.8% to 3rd/4th generation cephalosporins and carbapenems, respectively [19]. Previous reports suggest the increasing prevalence of MDR CR-Kp isolates in the Brazilian Amazon region [20,21,22]. As in line with the discussed data, the antimicrobial susceptibility features of *K. pneumoniae* isolates in our study demonstrated that all tested isolates were MDR (possible XDR), predominantly exhibiting combined resistance to 3rd/4th generation cephalosporins, carbapenems, and fluoroquinolones. Furthermore, even though the transmission of MDR *K. pneumoniae* between patients is an important mechanism for outbreaks occurrence, especially in ICUs [23,24], the presence of highly resistant isolates in non-ICU settings (13/23—56.5%) was observed. This points out the spread of these strains in different wards and/or different healthcare facilities, which may also be associated and contribute to disseminating antibiotic resistance markers, such as *bla*_NDM_ and *bla*_KPC_.

CR-Kp has been typically related to transmissible genetic elements added to carbapenemases genes, such as *bla*_NDM_ and *bla*_KPC_, which have been increasingly detected in different countries, emphasizing their worldwide dissemination [7,8,25,26]. In Brazil, KPC has become endemic and widely disseminated among *K. pneumoniae* from hospitals in all Brazilian regions [5,14,17,20,21,27,28,29,30]. Since 2013, NDM-producing isolates have been reported in different Brazilian regions and associated with a wide variety of bacterial species, including *Escherichia coli*, *Acinetobacter baumannii*, *A. nosocomialis*, *A. pittii, Enterobacter cloacae, Enterobacter hormaechei, and K. pneumoniae* [12,15,16,31,32,33,34,35]. Recently, Silva et al. [16], in a study evaluating 81 bacterial isolates from different regions, demonstrated that *K. pneumoniae* is responsible for the widespread of this carbapenemase in Brazilian hospitals, confirming more efficient dissemination compared with the *bla*_KPC_ variant. The study, however, was limited by not including isolates from the Brazilian Amazon region, evidencing the absence of data on NDM-producing *K. pneumoniae* isolates in the region. Interestingly, the present study revealed 23 CR-Kp isolates were harboring *bla*_NDM_ gene in nine different hospitals in the same region. This elevated frequency of NDM-producing CR-Kp has been only reported by Vivas et al. [15] in Sergipe (Northeast Brazil), where among the 147 investigated isolates, over 50.0% were *bla*_NDM_ carriers.

The NDM-7 variant was firstly described in *E. coli* and presented in its structure two amino acid substitutions compared to NDM-1 [36,37]. This variant has been related to infection by MDR microorganisms, predominantly in Asian countries, such as India, Japan, and China [38,39,40]. Despite being mainly associated with *E. coli* and *K. pneumoniae* isolates, NDM-7 has already been described in other members of Enterobacterales, reinforcing its ability to spread among different bacterial genera [41]. Furthermore, some studies have suggested more enzymatic hydrolysis activity against carbapenem, comparing NDM-7 to NDM-1 [42]. This study describes the alarming dissemination of NDM -1 and NDM-7 in association with *K. pneumoniae* HRCs in different healthcare institutions in northern Brazil, being the first detection of NDM-7 circulating in Latin America and Brazil.

Worryingly, the co-production and accumulation of genetic resistance determinants have become typically reported among highly resistant *K. pneumoniae* isolates. Certainly, most of the NDM-producing isolates co-harbor a broad variety of resistance mechanisms, such as CTX-M, SHV, KPC, VIM, and OXA-48, highlighting the increasing incidence of *bla*_NDM_ and *bla*_KPC_ co-producing strains, as reported in China, USA, Greece, India, Pakistan and Turkey [7,25,43,44,45,46,47,48]. In April 2021, Argentina’s National Antimicrobial Reference Laboratory was alerted to the emergence and spread of Enterobacterales producing different combinations of carbapenemases, especially during the first wave of the COVID-19 pandemic. Approximately one-third of the isolates received at this center (27.0%—52/196) were co-producers, with a combination of serine and MβLs, of which 60% had a combination of KPC and NDM [49]. In Brazil, the presence of over 20 *bla*_NDM_ and *bla*_KPC_ *K. pneumoniae* co-producing isolates, distributed among the Northeast, Southeast and South regions, have been described [12,15,27,31,50]. In the present study, four CR-Kp isolates were detected co-harboring *bla*_NDM_ and *bla*_KPC_. Three were recovered from patients hospitalized in the pediatric ICU at the same hospital (H3), suggesting that the persistence of high resistance levels strains in the environment and urgent strengthening of surveillance measures.

Investigations on the molecular epidemiology of *K. pneumoniae* isolates demonstrated that a small subset of successful HRCs is responsible for undermining antimicrobial therapy options, severe and poor prognosis infections, and nosocomial outbreaks globally. *K. pneumoniae* HCRs exhibit a high resistance degree, including resistance to 3rd/4th generation cephalosporins and carbapenems, and are remarkably effective reservoirs and vehicles for disseminating genetic resistance mechanisms, such as ESBLs and carbapenemases [7,10,51]. Molecular epidemiology analysis based on MLST revealed an absence of relationship among most of the evaluated *K. pneumoniae* strains, including the presence of nine distinct STs belonging to four CCs and predominance of MDR HRCs, which corroborates the hypothesis of epidemic multiclonal expansion of *K. pneumoniae* in Brazilian hospitals and globally.

Carbapenemase-producing clones are mostly associated with CC258, including STs 11, 101, 258, 340, 437, 512, 874, and 1264. ST11 and ST340/CC258 are globally distributed and have been detected harboring carbapenemases regardless of their type, and in Brazil, they are mostly associated with KPC-producing strains in almost all Brazilian regions [7,13,14,17,52,53,54,55]. ST1264 has emerged (and probably highly restricted) in China, causing bloodstream infection, and recently associated with ESBL-producing isolates [56,57]. Recently, several reports indicate ST340/CC258 has a critical role in expanding NDM-producing strains in Brazil, being associated with monoclonal outbreaks, colistin-resistant-isolates, and isolates co-harboring *bla*_NDM_ and *bla*_KPC_ genes [9,12,18,58]. Epidemic ST11 was the most prevalent in our study, found dispersed in five distinct hospitals in different periods, and interestingly present in pediatric settings at H3, co-harboring *bla*_NDM,_ and *bla*_KPC_, indicating endemicity and spread these resistance markers in this environment. ST340 was related to a single isolate recovered from a patient in the adult ICU at H3. To the best of our knowledge, the present study first provides data on the dissemination of NDM and NDM/KPC-producing *K. pneumoniae* associated with ST11/CC258 among clinical isolates in Brazil and corroborates the dissemination of ST340 NDM-producing strains in Brazil. Finally, our results also contribute to a better comprehension of the epidemiology of clinically important carbapenem resistance markers in the Brazilian territory and globally.

The HRC ST15/CC15 is usually related to CR-Kp carrying plasmid-borne MβLs in several locations in Europe, India, Nepal, Pakistan, and China, indicating a high capacity for the horizontal acquisition of resistance genes [6,10,59,60]. In Latin America, ST15/CC15 has been less frequently described than ST11 and ST340. However, it has demonstrated a concerning spread, with strains particularly harboring the *bla*_NDM_ gene and rarely associated with *bla*_KPC_ [54,61]. Since its first report in Brazil by Gonçalves et al. [17], ST15/CC15 has been detected in clinical and environmental samples, but with few reported NDM-producing strains in Porto Alegre (South region), Rio de Janeiro (Southeast), and Brasilia (Midwest region) cities [9,50,54,62]. According to our data, NDM-producing ST15/CC15 strains have been found circulating in four different hospitals since 2018 and mostly present adult settings, suggesting an early and rapid spread across Brazil.

*K. pneumoniae* ST392/CC147 has been reported related to nosocomial infection in various countries and described as an emerging clinically important HRC. Its endemicity has been related to the clonal spread of KPC-3-producing strains in Italy, VIM-producing strains in Grecia, while in Colombia, Mexico, and Iran with NDM-producing strains [63,64,65,66,67]. In Brazil, few reports revealed strains co-harboring *bla*_KPC_/*mcr-1* and *bla*_KPC_/*bla_OXA_* in Espírito Santo (Southern Brazil) and Tocantins (Northern Brazil), respectively [12,21]. In the present study, ST392 was detected in the pediatric clinic at H3, and, as far as we know, this is the first report of NDM-producing *K. pneumoniae* belonging to ST392/CC147 in Brazil.

Despite the non-HRCs being found related with the minority of NDM-producing *K. pneumoniae* isolates in our study, they were interestingly detected carrying the *bla*_NDM-7_ variant. ST1401 was first described in a human blood sample from Kuwait and has been reported in the USA and Mexico [68,69,70]. ST138/CC138 has been associated with KPC-2-producing and NDM-7-producing isolates in Brazil and Canada, respectively [14,71]. ST3449 was detected in China in an isolate harboring the *bla*_NDM_ gene [72]. An NDM/KPC-producing *K. quasipneumoniae* isolate belonging to ST3512 was to be causing bloodstream infection in Bahia State (Northeast Brazil) [11]. Wyres et al. [10] highlight that non-HCR may only cause localized problems; however, their emergence, spread, and persistence as HRC are influenced by several factors, which are mostly unknown. In this sense, our results draw attention to the importance of non-HRCs in the spread of NDM-7-producing strains at four hospitals (H1, H2, H7, and H8) in the Brazilian Amazon region, also been, to the best of our knowledge, the first report of ST1401, ST138, ST3449 and ST4398 carrying the *bla*_NDM-7_ gene in Latin America and Brazil.

## 4. Materials and Methods

### 4.1. Bacterial Isolates and Species Identification

The present cross-sectional study included 23 non-duplicated *K. pneumoniae* isolates stored at the Bacteriology and Mycology Section, Evandro Chagas Institute, a referral surveillance center located in the State of Pará, Brazilian Amazon region. From January 2018 to February 2021, *K. pneumoniae* isolates were obtained from several clinical sources of non-consecutive patients admitted at nine different hospitals in the region (H1-H9). Bacterial suspensions for each sample were prepared to match the 0.5 McFarland standard, followed by isolates identification on the automated VITEK-2 system (bioMérieux, Marcy l’Etoile, France) using the VITEK-2 card GN Test Kit Ref: 21341 for Gram-negative species identification.

### 4.2. Antimicrobial Susceptibility-Related Assays

AST was performed by broth microdilution on the automated VITEK-2 system (bioMérieux, Marcy l’Etoile, France) for 10 antimicrobial categories, including penicillins (ampicillin—AMP), penicillins + β-lactamase inhibitors (ampicillin-sulbactam—SAM), antipseudomonal penicillins + β-lactamase inhibitors (piperacillin + tazobactam—TZP), non-extended spectrum cephalosporins/2nd generation cephalosporins (cefuroxime—CXM), cephamycins (cefoxitin—FOX), extended-spectrum cephalosporins/3rd and 4th generation cephalosporins (ceftazidime—CAZ; ceftriaxone—CRO and cefepime—FEP), carbapenems (imipenem—IMP; meropenem—MEM and ertapenem—ETP), aminoglycosides (amikacin—AMK and gentamicin—GEN), fluoroquinolones (ciprofloxacin—CIP) and glycylcyclines (tigecycline—TGC). ATS was conducted following the manufacturer’s requirements, and as a result, isolates were classified as sensitive (S), intermediate (I), and resistant (R), based on breakpoints by the Clinical and Laboratory Standards Institute (CLSI), except for TGC, which was considered the FDA criteria [73,74].

Additionally, isolates were phenotypically categorized as MDR if resistant to ≥1 drug in ≥3 antimicrobial categories, according to criteria proposed by Magiorakos et al. [75]. Escherichia coli ATCC 25922 and Pseudomonas aeruginosa ATCC 27853 were used as quality control strains. Finally, all isolates exhibiting non-susceptibility to carbapenems (IMP, MER and/or ETP) were phenotypically tested for the presence of carbapenemase using the test of inactivation of carbapenem (mCIM/eCIM), following the CLSI recommendations [73].

### 4.3. Molecular Screening of β-Lactamase-Encoding Genes

Bacterial genomic DNA was extracted from overnight cultures, where a single *K. pneumoniae* colony was suspended in 300μL of distilled water and boiled at 95 °C for 10 min, followed by incubation at −20 °C for 15 min, and final centrifugation at 12,000 rpm for 7 min. The obtained DNA samples were stored at −20 °C until testing and used for all molecular assays. The molecular detection of β-lactamase-related genes (*bla*_KPC_, *bla*_NDM_, *bla*_IMP_, *bla*_VIM_, and *bla*_OXA-48_) was performed by PCR using the previously described primers and methodology [76]. PCR products were analyzed under UV light after electrophoresis on 1% agarose gel stained with SYBR™ Safe DNA gel stain (Life Technologies, Carlsbad, CA, USA). Definition of bla_NDM_ and bla_KPC_ subtypes was performed by direct sequencing of purified PCR products using the BigDye™ Terminator v3.1 Cycle Sequencing Kit (Life Technologies, Carlsbad, CA, USA) on an ABI Prism 3130 Genetic Analyzer (Applied Biosystems, Foster City, CA, USA). This was followed by the analysis of the obtained sequences at the BLAST search, available at the NCBI website (http://blast.ncbi.nih.gov/Blast.cgi (accessed on: 21 October 2021)) [76,77].

### 4.4. Genetic Diversity Assessment by MLST

Molecular typing by MLST was performed in accordance with a protocol previously described by Diancourt et al. [78], slightly modified by using universal sequencing primers. The seven housekeeping genes included in the scheme (gapA, infB, mdh, pgi, phoE, rpoB, and tonB) were amplified by PCR, followed by sequencing of reaction products using the BigDye™ Terminator v3.1 Cycle Sequencing Kit (Life Technologies, Carlsbad, CA, USA) on an ABI Prism 3130 Genetic Analyzer (Applied Biosystems, Foster City, CA, USA). Determination of allele profiles and sequence types (STs) was conducted by comparing the obtained sequences to the documented data at Klebsiella PasteurMLST database (https://bigsdb.web.pasteur.fr/Klebsiella/Klebsiella.html (accessed on 21 October 2021). PHYLOViZ 2.0 platform was used for data management and analysis of clonal complexes (CCs), which were defined by related ST clusters exhibiting variation in a single locus (single locus variants—SLV) [79].

## 5. Conclusions

In conclusion, the present study demonstrates the multiclonal dissemination of MDR *K. pneumoniae* HRCs producing NDM-1 and NDM-7 carbapenemases in different hospitals in Northern (Amazon region) Brazil. This highlights the need for reinforcement of surveillance and control measures for such strains. Epidemic HRCs ST11/CC258 and ST392/CC147 were firstly associated with NDM-producing strains in Brazil and the first detection of the NDM-7 variant in Latin America and Brazil. Finally, the concerning diversity of NDM variants associated with a diverse genetic background of *K. pneumoniae* strains suggests an early endemicity for this carbapenemase in the country, which may negatively impact healthcare and antimicrobial resistance scenarios locally and nationally.

## Figures and Tables

**Table 1:** Epidemiological and genotyping characteristics of the 23 NDM-producing *K. pneumoniae* isolates from different healthcare institutions, state of Pará, Brazilian Amazon region.

Isolate ID	Hospital	Ward	Clinical Specimen	Date	ST	CC	HRC	NDM Subtype
46956	(H1)	Pediatric clinic	Blood	20 August 2018	4398	Singleton	−	NDM-7
47098	(H2)	Adult ICU	Bronchoalveolar lavage	24 August 2018	15	15	+	NDM-1
47398	(H3)	Adult ICU	Urine	11 September 2018	340	258	+	NDM-1
49684	(H4)	Adult clinic	Urine	3 January 2019	15	15	+	NDM-1
50177	(H1)	Pediatric clinic	Blood	19 February 2019	1264	258	+	NDM-7
50467	(H5)	Adult ICU	Rectal swab	18 March 2019	15	15	+	NDM-7
50933	(H3)	Pediatric clinic	Urine	27 March 2019	392	147	+	NT
50937	(H3)	Pediatric clinic	Urine	15 April 2019	11	258	+	NDM-1
50938	(H6)	Adult clinic	Abdominal abscess secretion	10 April 2019	11	258	+	NDM-1
50942	(H2)	Adult clinic	Wound secretion	22 April 2019	15	15	+	NDM-1
51999	(H2)	Adult clinic	Urine	10 June 2019	11	258	+	NDM-7
51887	(H3)	Pediatric ICU	Tracheal secretion	14 May 2019	11	258	+	NDM-1
52012	(H2)	Adult ICU	Tracheal secretion	21 June 2019	3512	Singleton	−	NDM-7
54200	(H7)	Adult clinic	Urine	29 October 2019	3449	Singleton	−	NDM-7
56585	(H8)	Adult ICU	Rectal swab	29 May 2020	1401	1401	−	NDM-7
57319	(H3)	Pediatric ICU	Soft tissue secretion	20 November 2020	11	258	+	NDM-7
57351	(H1)	Pediatric clinic	Blood	1 February 2021	11	258	+	NDM-7
57352	(H9)	Adult clinic	Urine	24 January 2021	11	258	+	NDM-7
57387	(H3)	Pediatric ICU	Nasopharyngeal secretion	19 January 2021	11	258	+	NDM-7
57413	(H3)	Pediatric ICU	Rectal swab	28 January 2021	11	258	+	NDM-7
57414	(H3)	Adult clinic	Urine	26 January 2021	15	15	+	NDM-1
57420	(H9)	Adult ICU	Tracheal secretion	28 January 2021	11	258	+	NDM-7
57090	(H2)	Adult clinic	Peritoneal fluid	9 November 2020	138	138	−	NDM-7

ICU: Intensive care unit; ST: Sequence type; CC: Clonal complex; HRC: high risk clone; NT: not subtyped.

**Table 2:** Phenotypical and molecular susceptibility features of MDR *K. pneumoniae* isolates from different healthcare institutions, state of Pará, Brazilian Amazon region.

Isolate ID	MIC (µg/mL)															Carbapenemase Gene	mCIM	eCIM
	AMP	SAM	TZP	CXM	FOX	CAZ	CRO	FEP	ETP	IMP	MEM	AMK	GEN	CIP	TGC			
46956	>16	>16	>64	>32	>32	>32	>32	>32	>4	>8	>8	≤2	>8	≤0.25	≤0.5	*bla*_NDM-7_	+	+
47098	>16	>16	>64	>32	>32	>32	>32	>32	>4	≤0.25	4	8	>8	>2	≤0.5	*bla*_NDM-1_	+	+
47398	>16	>16	>64	>32	>32	>32	>32	>32	>4	>8	>8	16	>8	>2	2	*bla*_NDM-1_	+	+
49684	>16	>16	>64	>32	>32	>32	>32	>32	>4	>8	>8	16	≤1	>2	1	*bla*_NDM-1_	+	+
50177	>16	>16	>64	>32	>32	>32	>32	>32	>4	>8	>8	≤2	>8	>2	2	*bla*_NDM-7_	+	+
50467	32	32	128	64	64	64	64	32	4	16	16	≤2	16	≥4	1	*bla*_NDM-7_	+	+
50933	>16	>16	>64	>32	>32	>32	>32	>32	>4	>8	>8	≤2	>8	>2	>4	*bla*_NDM_ *	+	+
50937	>16	>16	>64	>32	>32	>32	>32	>32	>4	>8	>8	16	2	>2	2	*bla*_NDM-1_	+	+
50938	>16	>16	>64	>32	>32	>32	>32	>32	>4	>8	>8	16	≤1	>2	4	*bla*_NDM-1_/*bla*_KPC-2_	+	−
50942	>16	>16	>64	>32	>32	>32	>32	>32	>4	>8	>8	16	≤1	>2	2	*bla*_NDM-1_	+	+
51999	>16	>16	>64	>32	>32	>32	>32	>32	>4	>8	>8	>32	>8	>2	>4	*bla*_NDM-7_	+	+
51887	>16	>16	>64	>32	>32	>32	>32	>32	>4	>8	>8	16	≤1	>2	2	*bla*_NDM-1_/*bla*_KPC-2_	+	−
52012	>16	>16	>64	>32	>32	>32	>32	>32	>4	>8	>8	16	≤1	>2	>4	*bla*_NDM-7_	+	+
54200	>16	>16	>64	>32	>32	>32	>32	>32	>4	>8	>8	≤2	≤1	2	1	*bla*_NDM-7_	+	+
56585	>16	>16	>64	>32	>32	>32	>32	>32	>4	>8	>8	≤2	≤1	1	<0.5	*bla*_NDM-7_	+	+
57319	32	32	128	64	64	64	64	64	8	≥16	≥16	16	≥16	4	2	*bla*_NDM-7_	+	+
57351	>16	>16	>64	>32	>32	>32	>32	>32	>4	>8	>8	4	>8	>2	2	*bla*_NDM-7_	+	+
57352	>16	>16	>64	>32	>32	>32	>32	>32	>4	8	>8	≤2	>8	>2	2	*bla*_NDM-7_	+	+
57387	>16	>16	>64	>32	>32	>32	>32	>32	>4	>8	>8	≤2	≤1	>2	2	*bla*_NDM-7_/*bla*_KPC-2_	+	−
57413	>16	>16	>64	>32	>32	>32	>32	>32	>4	>8	>8	≤2	≤1	>2	2	*bla*_NDM-7_/*bla*_KPC-2_	+	+
57414	>16	>16	>64	>32	>32	>32	>32	>32	>4	>8	>8	16	≤1	>2	2	*bla*_NDM-1_	+	+
57420	>16	>16	>64	>32	>32	>32	>32	>32	>4	8	>8	≤2	8	>2	4	*bla*_NDM-7_	+	+
57090	>16	>16	>64	>32	>32	>32	>32	>32	>4	>8	>8	≤2	≤1	≤0.25	1	*bla*_NDM-7_	+	+

AMK: amikacin; AMP: ampicillin; CAZ: ceftazidime; CIP: ciprofloxacin; CRO: ceftriaxone; CXM: cefuroxime; ERT: ertapenem; FEP: cefepime; FOX: cefoxitin; GEN: gentamicin; IMP: imipenem; MEM: meropenem; MIC: minimal inhibitory concentration; SAM: ampicillin/sulbactam; TGC: tigecycline; TZP: piperacillin/tazobactam. * NDM subtyping could not be performed.

## Data Availability

All relevant data is presented within the manuscript.
